# Supplementary material for: Isothermal amplification and fluorescent detection of SARS-CoV-2 and SARS-CoV-2 variant virus in nasopharyngeal swabs
Source: PLoS One. 2021 Sep 17;16(9):e0257563. doi: 10.1371/journal.pone.0257563 (PMC8448339; doi:10.1371/journal.pone.0257563)
Supplement: S1 Fig — A. RT-qPCR using a CDC EUA N1 probe assay targeting purified genomic RNA from SARS-CoV-2 USA-WA1/2020. Log dilution series of purified genomic viral RNA underwent reverse transcription and cDNA was used in a CDC EUA N1 Probe Assay. Fluorescence data were collected and processed using a 40 cycle cut-off on an AriaMx Real-Time PCR System. B. Fluorescent RT-LAMP assay targeting purified SARS-CoV-2 USA-WA1/2020 RNA. Log dilution series of purified genomic RNA ranging from 108 to 10 copies of the N gene was added to fluorescent RT-LAMP reactions containing six LAMP primers (FIP/BIP, F3/B3, and LF/LB) and an SYBR-like fluorescent dye. Reactions were performed on an AriaMx Real-Time PCR System and fluorescence values were plotted against the elapsed reaction time (min) to generate the amplification curve. (DOCX) [file pone.0257563.s001.docx]

1. RT-qPCR CDC EUA N1 Probe Assay

1. Fluorescent RT-LAMP
