# Supplementary figures and images for: Isothermal amplification and fluorescent detection of SARS-CoV-2 and SARS-CoV-2 variant virus in nasopharyngeal swabs
Source: PLoS One. 2021 Sep 17;16(9):e0257563. doi: 10.1371/journal.pone.0257563 (PMC8448339; doi:10.1371/journal.pone.0257563)

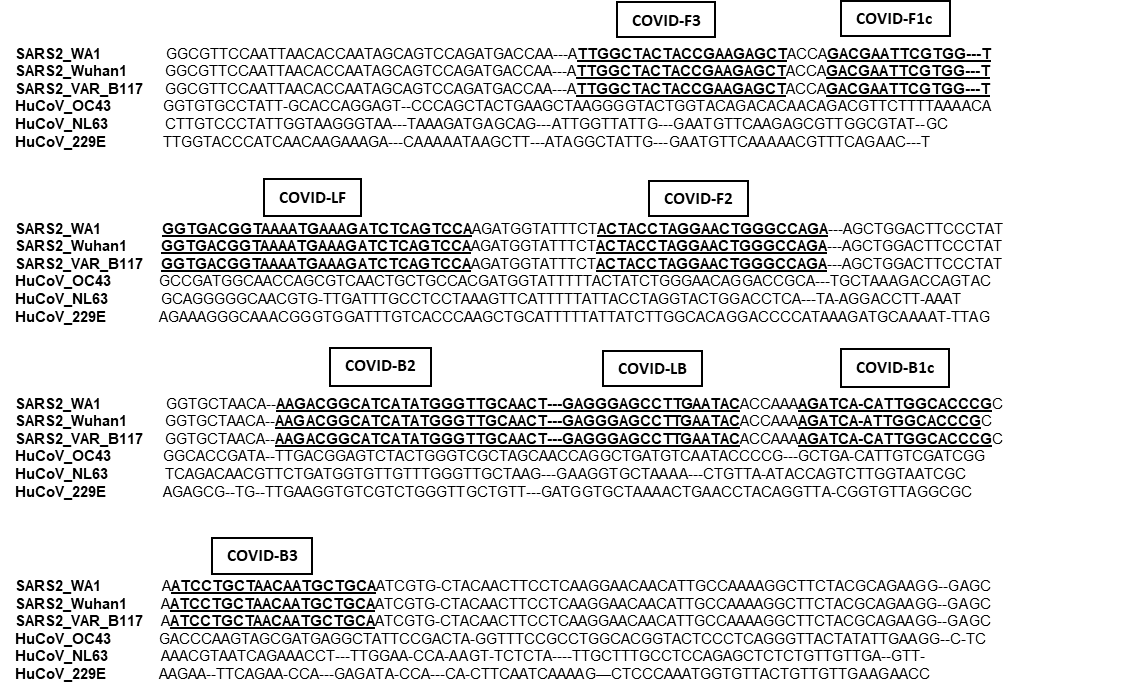

Supplement: S2 Fig — Nucleocapsid (N) gene multiple sequence alignment (T-Coffee) of SARS-CoV-2 strains: SARS2_WA1 (USA-WA1/2020 GenBank: MN985325.1), SARS2_Wuhan1 (Wuhan-Hu-1 GenBank: MN908947), SARS2_VAR_B117 (B.1.1.7 Alpha SARS-CoV-2/human/USA/SEARCH-5574-SAN/2020 GenBank: MW422255.1); and closely-related human coronavirus strains: HuCoV_OC43(GenBank: AY585228.1), HuCoV_NL63 (GenBank: AY567487.2), and HuCoV_229E (GenBank: AF304460.1) used in the study. Position of the six fluorescent RT-LAMP assay primers and their conservation (100%) among the wild-type and variant SARS-CoV-2 strains accounts for assay specificity for the variant strain, SARS2 B.1.1.7. (alpha). Divergence of the heterologous human coronavirus strain sequences results in no signal in the fluorescent RT-LAMP assay for these targets. (DOCX) [file pone.0257563.s002.docx]
