# Supplementary material for: Isothermal amplification and fluorescent detection of SARS-CoV-2 and SARS-CoV-2 variant virus in nasopharyngeal swabs
Source: PLoS One. 2021 Sep 17;16(9):e0257563. doi: 10.1371/journal.pone.0257563 (PMC8448339; doi:10.1371/journal.pone.0257563)
Supplement: S1 Table — N gene cloning primers, fluorescent RT-LAMP assay primers created using free Primer Explorer V software, and SARS-CoV-2 CDC EUA N1 RT-qPCR assay primers and probe. Numbering according to SARS-CoV-2 Wuhan-Hu-1 (GenBank: MN908947.3). (DOCX) [file pone.0257563.s004.docx]

**Supplemental Table 1. SARS-CoV-2 fluorescent RT-LAMP primers**

| **NAME** | **SEQUENCE (5’-3’)** | **Genome Position** |
| --- | --- | --- |
| SARS2N_F | ATGTCTGATAATGGACCCCAAAATC | 28274-28298 |
| SARS2N_R | TTAGGCCTGAGTTGAGTCAGCAC | 29511-29533 |
| COVID-F3 | TGGCTACTACCGAAGAGCT | 28525-28543 |
| COVID-B3 | TGCAGCATTGTTAGCAGGAT | 28722-28741 |
| COVID-LF | GCCATTTTACTTTCTAGAGTCAGGT | 28567-28591 |
| COVID-LB | ACTGAGGGAGCCTTGAATAC | 28676-28695 |
| COVID-FIP (F1c) | GACGAATTCGTGGTGGTGA | 28548-28566 |
| COVID-FIP (F2) | TCTGGCCCAGTTCCTAGGTAGT | 28605-28626 |
| COVID-BIP (B1c) | CGGGTGCCAATGTGATCT | 28702-28719 |
| COVID-BIP (B2) | AGACGGCATCATATGGGTTGCA | 28654-28675 |
| 2019-nCoV_ N1 Forward | GACCCCAAAATCAGCGAAAT | 28287-28306 |
| 2019-nCoV_N1 Reverse | TCTGGTTACTGCCAGTTGAATCTG | 28335-28358 |
| 2019-nCoV_N1 Probe | ACCCCGCATTACGTTTGGTGGACC | 28309-28332 |

**Supplemental Table 1.** N gene cloning primers, fluorescent RT-LAMP assay primers created using free Primer Explorer V software, and SARS-CoV-2 CDC EUA N1 RT-qPCR assay primers and probe. Numbering according to SARS-CoV-2 Wuhan-Hu-1 (GenBank: MN908947.3).
