# Supplementary material for: Isothermal amplification and fluorescent detection of SARS-CoV-2 and SARS-CoV-2 variant virus in nasopharyngeal swabs
Source: PLoS One. 2021 Sep 17;16(9):e0257563. doi: 10.1371/journal.pone.0257563 (PMC8448339; doi:10.1371/journal.pone.0257563)
Supplement: S1 Raw images — (PDF) [file pone.0257563.s005.pdf]

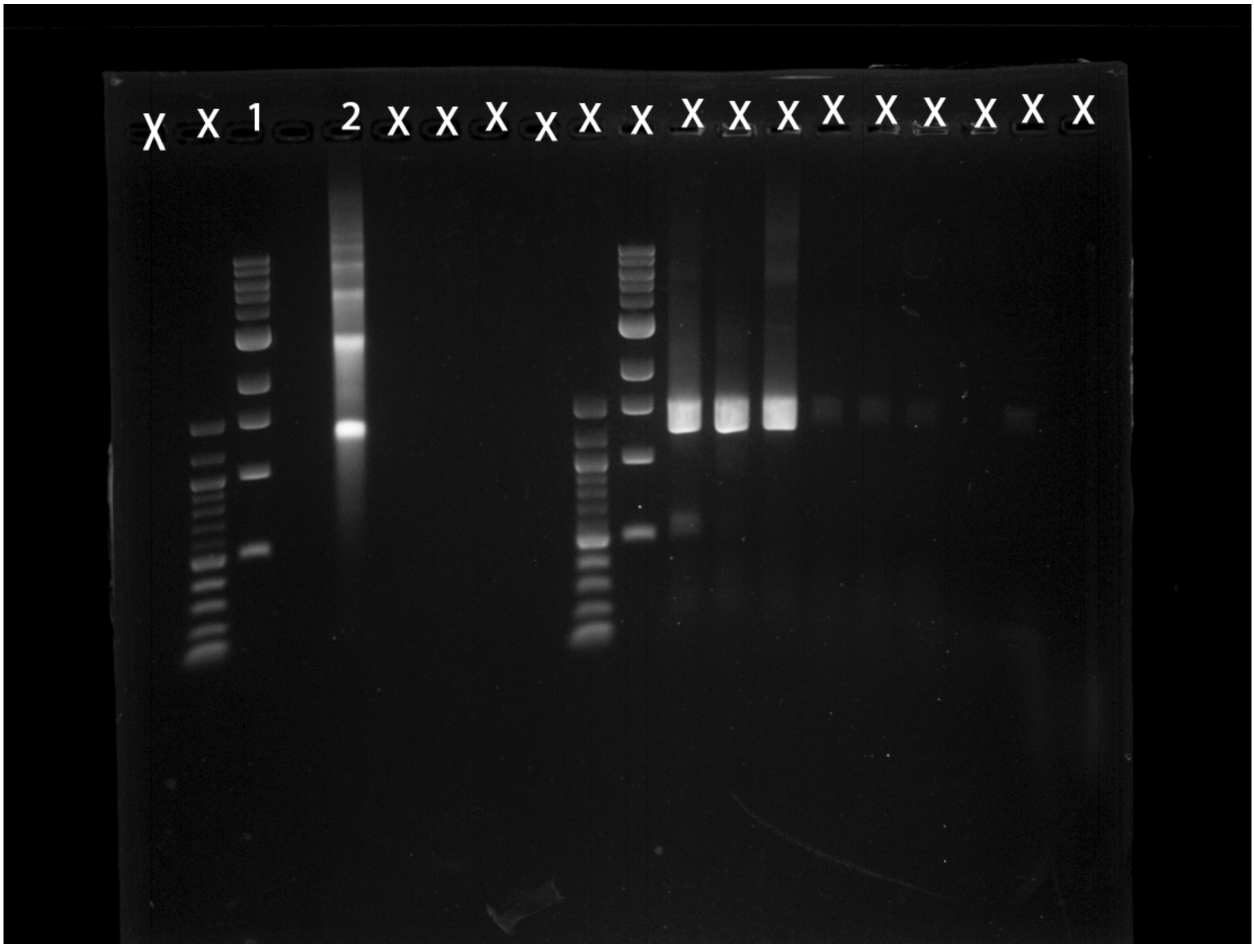

SARS-CoV-2 N gene IVT RNA after DNase treatment and purification. Non-denaturing agarose gel, stained with ethidium bromide. Image captured by UV transilluminator on ProteinSimple Imager machine. Figure 1, panel C was generated from this original image.

Lane 1: molecular weight marker

Lane 2: SARS-CoV-2 IVT RNA
