# Supplementary material for: Isothermal amplification and fluorescent detection of SARS-CoV-2 and SARS-CoV-2 variant virus in nasopharyngeal swabs
Source: PLoS One. 2021 Sep 17;16(9):e0257563. doi: 10.1371/journal.pone.0257563 (PMC8448339; doi:10.1371/journal.pone.0257563)
Supplement: Supplementary file 5 [file pone.0257563.s003.docx]

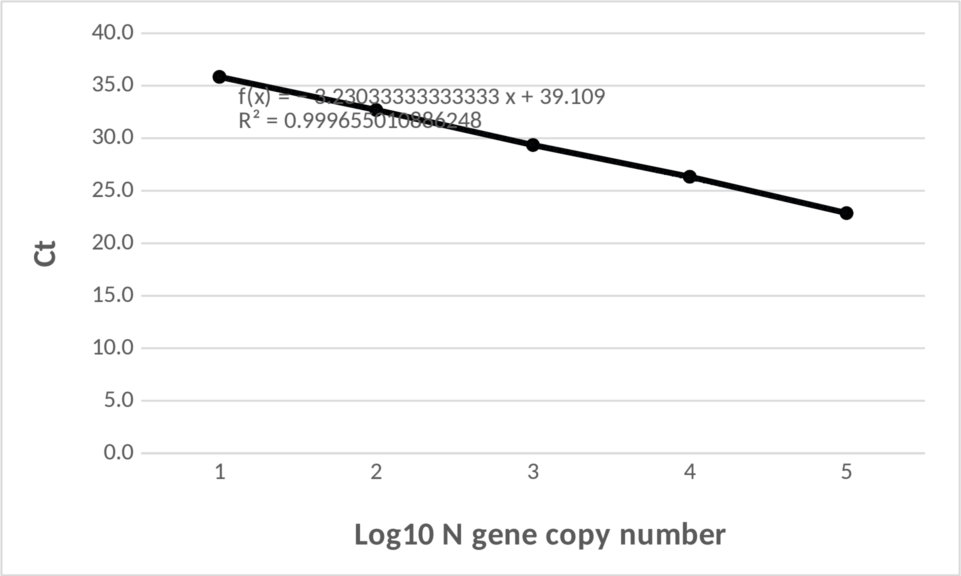

40.0
35.0
30.0
25.0
20.0
15.0
10.0
5.0
0.0
Ct
f(x) = − 3.23033333333333 x + 39.109
R² = 0.999655010886248
1
2
3
4
5
Log10 N gene copy number
