# Supplementary material for: Isothermal amplification and fluorescent detection of SARS-CoV-2 and SARS-CoV-2 variant virus in nasopharyngeal swabs
Source: PLoS One. 2021 Sep 17;16(9):e0257563. doi: 10.1371/journal.pone.0257563 (PMC8448339; doi:10.1371/journal.pone.0257563)
Supplement: Attachment — Submitted filename: Reviewer comments Isothermal Amplification and Fluorescent Detection of SARS-CoV-2.docx [file pone.0257563.s006.docx]

“Isothermal Amplification and Fluorescent Detection of SARS-CoV-2 and SARS-CoV-2 Variant Virus in Nasal Swabs".

The authors have documented the development of a Fluorescent LAMP detection method that can be completed in 30 minutes from minimally pre-processed nasal swabs. It shows to be faster and sensitive enough, when compared to RT-PCR, the globally accepted standard technique for detecting SARS-CoV-2.

Repeated information in Introduction second paragraph: “reduced cost, speed…sensitivity”. It can be consolidated.

Materials and Methods, RT-LAMP reactions, second paragraph:

Please specify which kind of sample in: “For patient sample fluorescent…”

The micro units must be spelled correctly as symbol, not “u”.

Same paragraph as above, word specificity is misspelled.

Fourth paragraph: …”QPCR machine”…, use thermal cycler or thermocycler.

Tables 2 through 4 are not cited in the main text. This needs to be corrected.

Discussion. One important issue addressed in this paper is that the proposed method has the capacity to detect different SARS-CoV-2 variants. It would help the paper to (briefly) discuss why that is technically possible.
